# Cross-sectional assessments of participants’ characteristics and loss to follow-up in the first Opioid Substitution Therapy Pilot Program in Kabul, Afghanistan

**DOI:** 10.1186/s12954-015-0062-1

**Published:** 2015-09-04

**Authors:** Horacio Ruiseñor-Escudero, Alexander Vu, Andrea L Wirtz, Itziar Familiar-Lopez, Mark Berry, Iliassou Mfochive, Cyrus Engineer, Ahmad Farhad, Senop Tschakarjan, Ernst Wisse, Feda M Paikan, Gilbert Burnham

**Affiliations:** Department of Psychiatry, Michigan State University, College of Ostheopathic Medicine, 965 E Fee Hall Suite A227, Lansing, MI 48824 USA; Department of International Health, Johns Hopkins Bloomberg School of Public Health, 615 N Wolfe Street, Baltimore, MD 21205 USA; Department of Emergency Medicine, Johns Hopkins University, School of Medicine, 5801 Smith Avenue, Suite 3220, Baltimore, MD 21205 USA; Department of Epidemiology, Johns Hopkins Bloomberg School of Public Health, Center for Public Health and Human Rights, 615 N Wolfe Street, Baltimore, MD 21205 USA; Department of Epidemiology, Johns Hopkins Bloomberg School of Public Health, 615 N Wolfe Street, Baltimore, MD 21205 USA; Medecins du Monde, Kabul, Afghanistan; Medecins du Monde, 63 rue de Marcadet, Paris, 75018 France; National AIDS Control Program, Kabul, Afghanistan; Department of Interprofessional Health Studies, Towson University, Baltimore, MD 21252 USA

**Keywords:** Opiate substitution therapy, Heroin, Methadone, Injecting drug use, Afghanistan

## Abstract

**Background:**

Kabul has over 12,000 people who inject drugs (PWID), most of them heroin users, and opioid substitution therapy has recently been introduced as an effective method to reduce opioid use. We aimed to evaluate a pilot Opioid Substitution Therapy Pilot Program (OSTPP) in Kabul, Afghanistan, particularly to (1) describe characteristics of the participants enrolled in the program and (2) identify factors associated with client retention in the OSTPP.

**Findings:**

Two cross-sectional surveys evaluated participants attending the OSTPP at baseline (*n* = 83) and 18 months after (*n* = 57). Questionnaires assessed socio-demographic, drug use behavior, and general and mental health factors. After 18 months, 57 participants remained in the OSTPP. Participants lost to follow-up were younger (*p* < 0.01) and married (*p* < 0.01) and had no family contact (*p* < 0.01). Participants at 18 months reported no criminal activity in the last month and only two (3.5 %) reported heroin use in the last month, constituting significant decreases from baseline.

**Conclusions:**

While preliminary results are promising, further evaluation is needed to determine the feasibility of implementing OSTPP in this setting and effectiveness in reducing injection risk behaviors in Afghanistan.

## Introduction

In 2012, it was estimated that over 12,000 PWID were living in Kabul alone [1]. Between 2008 and 2011, Afghanistan was one of only five countries in the world to report an increase in the prevalence of injecting drug use [2], likely attributable to broad structural factors including poor socioeconomic conditions, the increasing number of returning refugees, high-opium production, and new trafficking routes [3, 4]. As elsewhere, in Afghanistan, injecting drug use has been associated with hepatitis C virus (HCV) and HIV transmission [5, 6] and with substantial social implications, such as loss of employment and productivity [7].

Opioid substitution therapy (OST) is an evidence-based pharmacological intervention to treat opioid dependence [8]. OST replaces the use of illicit opiates, indirectly reduces injecting frequency, and improves health and social outcomes and evidence further suggests that OST may reduce HIV incidence [9, 10].

In February 2010, Medecins du Monde (MdM), with support from the Afghan Ministry of Public Health (MoPH) and the World Bank, implemented the first OST pilot program (OSTPP) in Kabul, Afghanistan. In 2012, the Johns Hopkins University (JHU) evaluated the OSTPP in collaboration with MdM and the Afghan National AIDS Control Program (NACP). This analysis aimed to (1) describe the characteristics of the participants enrolled in the program and (2) identify factors associated with client retention.

## Methods

### Setting and participants

The OSTPP clinic was located in central Kabul. Daily clinical visits were required of participants for daily methadone dosing. Ancillary services included psychosocial therapy, basic medical services, recreational activities, and educational programs. MdM enrolled active injecting drug users giving priority to those with poorer medical assessments. Participants were invited by MdM’s out-reach services to enroll into the OSTPP. Edibility criteria for baseline included providing consent and currently injecting heroin and for endline assessment included being an enrolled participant of the OSTPP, male, ≥18 years, and providing verbal informed consent.

### Data collection and procedures

Data are derived from two cross-sectional surveys conducted at enrollment (baseline) and at endline, 18 months later. After completing the verbal informed consent process, participants at baseline assessment and endline were surveyed using a structured, interviewer-administered questionnaire that included socio-demographic characteristics, injecting behaviors, criminal history, social integration, and general and mental health topics. Data were collected by trained MdM interviewers at baseline and by trained JHU interviewers at endline. All questions were developed in English, translated into Dari, and back-translated by a certified translator. No participation incentive was provided.

The study was approved by the Institutional Review Boards of the Afghan MoPH and Johns Hopkins Bloomberg School of Public Health.

### Measures

Mental health was assessed prior to enrollment by self-report of depressive symptoms using a 10-item measure developed by MdM for patient intake, which included psychotic symptoms (five items) and post-traumatic stress disorder (PTSD) symptoms (five items). Responses to each symptom domain were recorded as “Yes” (present) or “No” (absent) and added to obtain total symptom counts and for each domain, separately. Cronbach’s alpha was 0.93. General health status was self-reported using a 10-point Likert scale, with higher scores indicating better self-reported health.

Measures of substance use behaviors included heroin use after enrollment into the OSTPP, frequency of injection in the last month (once a day/daily vs. less than daily for analysis), history of imprisonment, and frequency of contact with family members in the past month and were categorized as binary responses. HIV 1/2 and HCV-Ab testing were conducted by MDM using Afghan MoPH guidelines.

### Data analysis

Secondary data analysis was conducted with data from baseline participants and endline participants. MdM collects participant’s data anonymously, prohibiting identification of the participants who were later enrolled into the program. Descriptive statistics summarized socio-demographic characteristics, health status, heroin use, and injecting characteristics. Attrition analysis was conducted to compare participant characteristics of those who remained enrolled in the program and those lost to follow-up. Statistical comparisons were made using chi-square tests for binary and categorical data and independent *t*-tests for continuous variables. STATA version 12 (College Station, TX 2012) was used for statistical analyses.

## Findings

**Fig. 1:**
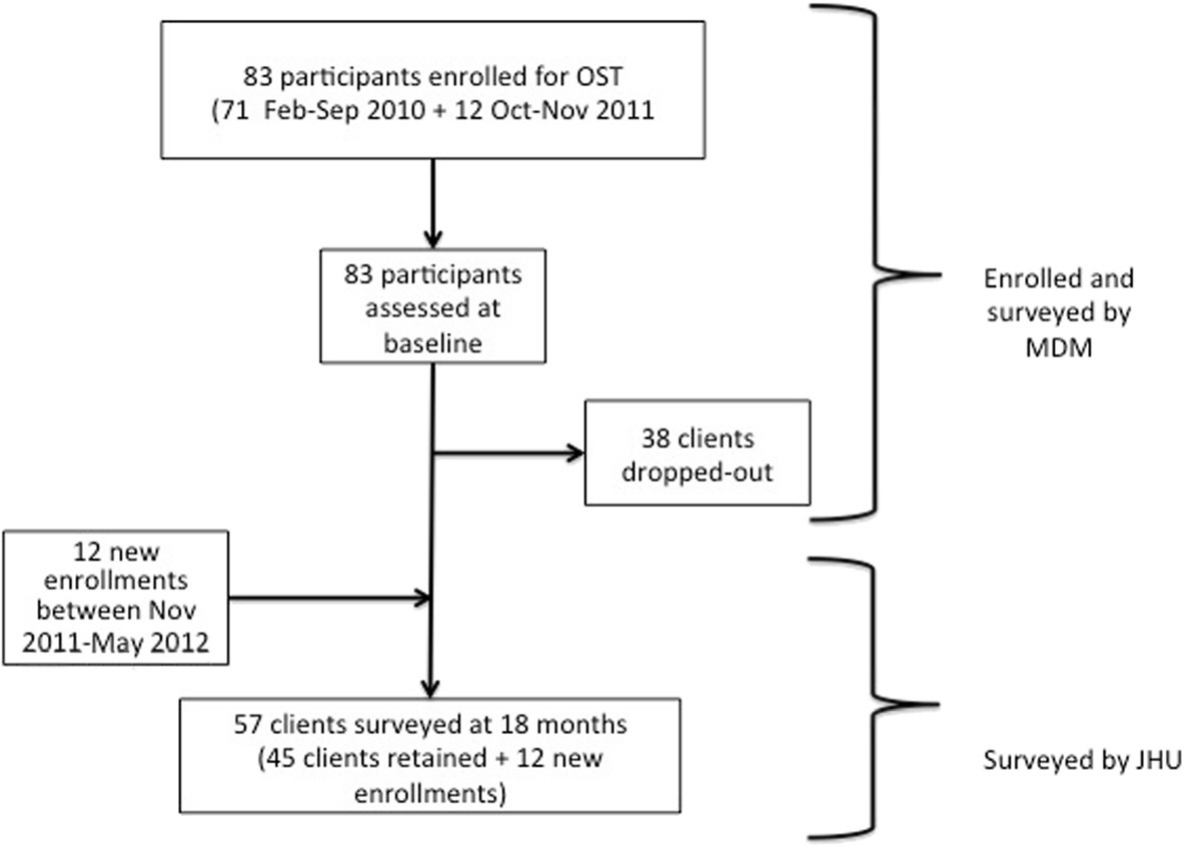
Participants enrolled and surveyed in the OSTPP between February 2010 and May 2012. Eighty-three participants were initially enrolled and surveyed by MdM, 38 dropped-out, and 45 were retained. After 18 months, 57 clients were surveyed by JHU

Overall, between February 2010 and May 2012, 95 participants were enrolled in the OSTPP. The initial number of program participants was restricted by NACP and the Ministry of Counter Narcotics to 71 participants, who were enrolled between February and September 2010. An additional 12 participants were enrolled between October 2011 and Nov 2011, totaling 83 participants with a baseline evaluation. An additional 12 participants were enrolled between November 2011 and May 2012, resulting in a total of 57 clients who were surveyed at endline by JHU (Fig. 1). All participants were male and with a mean age of 32.2 years (SD = 7.8) at baseline. The mean daily methadone dose was of 131 mg/day to all participants (range = 30–270 mg/day). Of the 83 clients who were initially enrolled and surveyed by MdM, 38 clients were lost to follow-up (45.7 %) and 45 clients were retained (54.2) and at 18 months (Table 1).

**Table 1:** Baseline socio-demographic and drug use characteristics of participants enrolled, lost to follow-up, and retained in the OSTPP in Kabul, Afghanistan

Characteristic	Total baseline participants (*N* = 83)	Lost to follow-up (*N* = 38)	Baseline participants retained by endline (*N* = 45)	*p* value
	*n* (%)	*n* (%)	*n* (%)	
Age (years)				
18–24	13 (15.7)	11 (29.0)	2 (4.4)	<0.01
25–29	23 (27.7)	10 (26.3)	13 (28.9)	0.77
30–34	17 (20.5)	7 (18.4)	10 (22.2)	0.51
35+	30 (36.1)	10 (26.3)	20 (44.4)	0.01
Marital status^a^				
Single	43(51.8)	14 (37.8)	29 (64.4)	<0.01
Married/engaged	37 (44.5)	21 (56.8)	16 (35.6)	
Family contact in the past month				
No contact	21 (25.3)	16 (42.1)	5 (11.1)	<0.01
Less than daily	16 (19.3)	7 (18.4)	9 (20.0)	
Daily	46 (55.4)	15 (39.5)	31 (68.9)	
General health score mean (SD) (range: 1–10)	5.1 (2.1)	5.2 (2.4)	5.0 (1.8)	0.67
Mental health symptoms (range: 1–20)				
Total number of symptoms, mean (SD)	6.5 (3.7)	6.7 (4.4)	6.2 (2.9)	0.62
Depressive symptoms mean(SD)	4.3 (2.4)	4.5 (2.5)	4.1 (2.3)	0.42
PTSD symptoms mean (SD)	2.7 (1.6)	1.2 (1.5)	1.5 (1.6)	0.34
Psychotic symptoms mean (SD)	1.5 (1.4)	1.3 (1.7)	0.38 (1.1)	<0.01
Frequency of heroin use^b^				
Once a week—less than daily	1 (1.2)	1 (2.6)	0 (0)	0.05
Daily	79 (95.2)	36 (94.7)	43 (95.6)	
No response	3 (3.6)	1 (2.6)	2 (4.4)	
Ever injected drugs				
Yes (not in the past month)	34 (41.0)	15 (39.5)	19 (42.2)	0.67
Yes (past month)	36 (43.4)	15 (38.5)	21 (46.7)	
No response	13 (15.7)	8 (2.2)	5 (2.2)	
Age first heroin use mean (SD)	22.8 (5.8)	21.1 (5.3)	24.4 (6.3)	0.01
Ever treated for substance use				
Yes	62 (74.7)	29 (76.3)	33 (73.3)	0.42
No	19 (22.9)	9 (23.7)	10 (22.2)	
No response	2 (2.4)	0 (0)	2 (4.4)	
Ever in prison	43 (51.8)	21 (55.3)	22 (48.9)	0.43
HIV positive^c^	5 (6.0)	2 (5.2)	3 (6.6)	0.59
HCV positive^c^	50 (60.2)	22 (57.9)	28 (62.2)	0.59

^a^One client refused to answer

^b^Heroine use includes injection, eating, sniffing, or smoking heroin

^c^HIV and HCV testing were conducted by MdM during completion of the baseline questionnaire

^d^Proportion presented in this table may not add to 100 % due to rounding

Reasons for loss to follow-up included voluntary withdrawal (*n* = 9, 20 %), imprisonment (*n* = 8, 17.8 %), death (*n* = 5, 11.1 %), migration (*n* = 3, 6.7 %), and other causes (*n* = 20, 44.4 %). Relative to those lost to follow-up, participants who remained enrolled were more likely to be older (*p* = 0.01), single (*p* < 0.01), report daily family contact in the past month (*p* < 0.01), older age at first heroin use (*p* = 0.01), and fewer number of psychotic symptoms (*p* < 0.01) (Tables 1 and 2).

**Table 2:** Distribution of characteristics among participants at baseline and endline evaluation of an OSTPP, in Kabul, Afghanistan

Characteristics	Baseline participants (*N* = 83)	Endline participants (*N* = 57)	*p* value
	*n* (%)	*n* (%)	
Mental health symptom median (SD)	6 (24.1)	3.0 (3.7)	<0.001
General health symptom median (SD)	5.0 (2.1)	8.0 (1.7)	<0.001
Current heroin use (any type)	83 (100)	35 (61.4)^a^	<0.001
Correct HIV knowledge	50 (60.2)	43 (75.4)	0.07
Has source of income	17 (20.5)	48 (84.2)	<0.001
History of criminal activity	51 (61.5)	0 (0)	<0.001
Any family contact	21 (25.3)	41 (71.9)	<0.001

^a^At endline, 15 participants (26.3 %) reported injecting heroin at some point after enrollment. Two participants (3.5 %) reported injecting in the last month

^b^All six characteristics presented in column one reflect the characteristic reported before enrollment vs. characteristic reported after enrollment

Relative to baseline participants, endline participants had better mental health (*p* < 0.001) and general health scores (*p* < 0.001), reported less heroin use (*p* < 0.001), less criminal activity (*p* < 0.001), improved family contact (*p* < 0.001), and a source of income (*p* < 0.001) (see Table 2).

## Discussion

This is the first report on the retention, characteristics, and behaviors of participants in the only OSTPP in Kabul, Afghanistan. Results suggest that even under challenging circumstances, participants who remained active in the OSTPP reported improved general and mental health status, decreased heroin use and criminal activity, and improved family contact.

Program retention was relatively high (over 50 %) and comparable to programs in high-income countries, despite severe disruptions in methadone provision derived from a non-functioning importation policy during the OSTPP implementation [11]. This disruption occurred between April and May 2011 and has been associated with the death of 4 clients [4, 12]. This suggests that even in conditions of substantial challenges, including restricted clinic working hours, required daily treatment visits, methadone stock-outs, and general instability and physical danger in Kabul, scale-up of methadone-based OST may be feasible in Kabul and could promote a positive change in specific behaviors and characteristics of participants on treatment.

Several baseline factors associated with loss to follow-up and retention were identified. Age at enrollment was related to retention; participants lost to follow-up were younger than those who remained enrolled. Loss of young participants is a critical barrier to overcome: young people are more vulnerable to initiation of substance use and most new HIV infections are reported among those 18–30 years old [13]. Understanding the needs and social situation of young people will facilitate OST development and implementation across this age group and may mitigate their risk of HIV and HCV infection [9].

Retention in the OSTPP was more likely among those with more family contact, suggesting that participants may benefit from family engagement in a treatment program [14]. Contrary to expectations, single participants at baseline were more likely to continue in the program, which may reflect having fewer social and economic needs than married participants and, thus, having more freedom to attend the clinic on a daily basis. The provision of take-home methadone, which has shown promise in other OST programs may help improve program retention by addressing the needs of participants who are married or employed [15–18].

The majority of participants retained in OSTPP reported reductions in heroin use, which is comparable to estimates reported in other countries [19]. By stopping heroin use, reductions in needle sharing and injecting frequency are expected, translating into fewer opportunities for HIV and HCV transmission and acquisition [20, 21], as well as other social benefits such as decreased criminal activity [22]. However, for those who continue to use heroin but at a lower rate, the effect of OSTPP on HIV and HCV infection might be marginal given the limited options and access to other harm reduction services [4, 23].

Findings should be viewed in light of several limitations. Due to restrictions on the number of enrolled participants, the overall evaluation’s sample size was small. Almost half of the baseline participants were lost to follow-up, decreasing the study’s power and directly affecting the generalizability and our capacity to make inferences. Participants in this study tended to be older, single, report ever being in prison, and report lower levels of employment than the wider population of injecting drug users in Kabul, thus further limiting the generalizability of our findings to the wider population of PWID in the city [1]. Interpretation of the results that compare the 83 baseline participants to the 57 endline participants must consider the fact that 12 participants did not provide baseline data. This may limit our capacity to accurately understand the factors associated with retention and OST outcomes. The OSTPP assessment was carried out within an existing program, lacking a control group and randomization. Baseline and endline data analysis reflect group changes and not individual change. Another potential bias was social desirability bias that is inherent in all socio-behavioral studies, particularly those that address illegal behaviors. The mental health questions used as part of the evaluation have not been validated for use among PWID. Future research should include validated mental health assessments that can better characterize the mental health problems of PWID. Reasons for recidivism should be identified with the aim of improving the program’s performance in maintaining heroin cessation. Finally, we have no information on participants’ participation in ancillary services. This information would inform on the impact of these services on participants’ retention.

## Conclusion

Despite limitations, findings suggest that OST in Afghanistan may be feasible in Kabul and could have positive impacts on participant behavior, supporting recommendations for use in other urban centers, such as Herat, where opioid use is prevalent [1]. This study provides preliminary data for future research and trials of optimal treatment and harm reduction programs for this setting.

## Abbreviations

HCV: hepatitis C Virus
MdM: Medecins du Monde
MoPH: Afghan Ministry of Public Health
NACP: Afghan National AIDS Control Program
OST: opioid substitution therapy
OSTPP: Opioid Substitution Therapy Pilot Program
PTSD: post-traumatic stress disorder
